# Supplementary material for: Epidemiological cut-off values for a 96-well broth microdilution plate for high-throughput research antibiotic susceptibility testing of M. tuberculosis
Source: Eur Respir J. 2022 Oct 13;60(4):2200239. doi: 10.1183/13993003.00239-2022 (PMC9556810; doi:10.1183/13993003.00239-2022)
Supplement: Supplementary file 1 [file ERJ-00239-2022.Shareable.pdf]

EUROPEAN RESPIRATORY JOURNAL
TASK FORCE REPORT
THE CRyPTIC CONSORTIUM

SHAREABLE PDF

Epidemiological cut-off values for a 96-well broth microdilution plate for high-throughput research antibiotic susceptibility testing of *M. tuberculosis*

The CRyPTIC Consortium[1]

[1]For a list of all members of the CRyPTIC Consortium and their affiliations, please see the section at the end of this article.

Corresponding author: Philip W. Fowler (philip.fowler@ndm.ox.ac.uk)

Check for updates

Shareable abstract (@ERSpublications)
International tuberculosis (TB) consortium proposes epidemiological cut-off values for a 96-well broth microdilution plate containing 13 anti-TB drugs using a dataset of 15 211 *M. tuberculosis* samples https://bit.ly/3vpOzIk

Cite this article as: The CRyPTIC Consortium. Epidemiological cut-off values for a 96-well broth microdilution plate for high-throughput research antibiotic susceptibility testing of *M. tuberculosis*. *Eur Respir J* 2022; 60: 2200239 [DOI: 10.1183/13993003.00239-2022].

This single-page version can be shared freely online.

Copyright ©The authors 2022.

This version is distributed under the terms of the Creative Commons Attribution Licence 4.0.

Received: 27 Oct 2021
Accepted: 22 Feb 2022

Abstract

Drug susceptibility testing of *M. tuberculosis* is rooted in a binary susceptible/resistant paradigm. While there are considerable advantages in measuring the minimum inhibitory concentrations (MICs) of a panel of drugs for an isolate, it is necessary to measure the epidemiological cut-off values (ECOFF/ECVs) to permit comparison with qualitative data. Here we present ECOFF/ECVs for 13 anti-tuberculosis compounds, including bedaquiline and delamanid, derived from 20 637 clinical isolates collected by 14 laboratories based in 11 countries on five continents. Each isolate was incubated for 14 days on a dry 96-well broth microdilution plate and then read. Resistance to most of the drugs due to prior exposure is expected and the MIC distributions for many of the compounds are complex, and therefore a *phenotypically* wild-type population could not be defined. Since a majority of samples also underwent genetic sequencing, we defined a *genotypically* wild-type population and measured the MIC of the 99th percentile by direct measurement and *via* fitting a Gaussian using interval regression. The proposed ECOFF/ECVs were then validated by comparing with the MIC distributions of high-confidence genetic variants that confer resistance and with qualitative drug susceptibility tests obtained *via* the Mycobacterial Growth Indicator Tube (MGIT) system or Microscopic-Observation Drug Susceptibility (MODS) assay. These ECOFF/ECVs will inform and encourage the more widespread adoption of broth microdilution: this is a cheap culture-based method that tests the susceptibility of 12–14 antibiotics on a single 96-well plate and so could help personalise the treatment of tuberculosis.
